# Editorial: The socio-economic burden of food allergy: from households to healthcare systems

**DOI:** 10.3389/falgy.2025.1608998

**Published:** 2025-04-28

**Authors:** Jennifer L. P. Protudjer, Lucy A. Bilaver

**Affiliations:** ^1^ Department of Pediatrics and Child Health, Max Rady College of Medicine, Rady Faculty of Health Sciences, University of Manitoba, Winnipeg, MB, Canada; ^2^ Children’s Hospital Research Institute of Manitoba, Winnipeg, MB, Canada; ^3^ Department of Food and Human Nutritional Sciences, Faculty of Agricultural and Food Sciences, University of Manitoba, Winnipeg, MB, Canada; ^4^ Institute of Environmental Medicine, Karolinska Institutet, Stockholm, Sweden; ^5^ Department of Pediatrics, Northwestern University, Chicago, IL, United States; ^6^ Center for Food Allergy and Asthma Research, Institute for Public Health and Medicine, Northwestern University, Chicago, IL, United States

**Keywords:** food allergy, health economics, financial burden, households, health disparities

**Editorial on the Research Topic**
The socio-economic burden of food allergy: from households to healthcare systems

Food allergy is expensive. Both affected households and the healthcare system are saddled with costs relating to this condition that involves careful dietary management, and constant emergency preparedness. Our Research Topic, titled *The Socio-economic Burden of Food Allergy: From Households to Healthcare Systems*, included articles that provided unique insights into food allergy costs, as well as some discussion on efforts to support those most affected.

From a household perspective, food allergy costs are primarily driven by the cost of food. This was true prior to the pandemic, but has been further exacerbated by the near-constant increases in food prices since 2020. Golding et al. reported on *Changes in food-related costs during the COVID-19 pandemic among families managing food allergy.* Unsurprisingly, the authors identified that costs did indeed increase. However, increases varied by household income. Direct cost increases were about double in higher income households, compared to lower income households, with a difference of about $114 Canadian dollars, or about 2% of the median household income for the sample. Importantly, these costs do not represent household spending on food, but rather the increase in household spending on food in the early days of the pandemic. Both lower and higher income households reported increased indirect costs dedicated to food preparation.

From a healthcare system perspective, Ahlstedt et al. reported on *Changes in epinephrine dispensings and allergy hospitalizations in Sweden in the years following the removal of auto-injector co-payments.* This is a follow-up paper to their original article, which explored EAI dispensings in the years prior to, and the two years following the removal of co-payments (1). In the present study, the authors identified that, despite no co-payments, EAI dispensings remained stable from 2018 to 2022, albeit with more dispensings for children ages 5–18 years than adults. Children ages 0–4 years had the lowest dispensings and highest rates of hospitalizations, albeit differences which did not statistically differ from older ages. Taken collectively, this study, which was based on national registry data, provides evidence that EAI dispensings are more common for children than adults, but that the removal of co-payments does not appear to contribute to excess levels of EAI dispensings.

With the ever-increasing costs of food, some efforts have been made to support those families most affected. Cow's milk allergy is one of the most common food allergies in young children, as well as being the most burdensome (2). To address this duality, in another paper by Golding et al., the authors created *An investigation of novel milk allergy-friendly food supplement program.* Over six months, these authors delivered an allergen-friendly food supplement program to low-to-middle income families. While families’ food costs increased and food preparation costs decreased over the study period, these differences were not statistically significant relative to baseline. Of note, costs related to lost work and school due to food allergy, significantly decreased, suggesting that an allergy-friendly food supplement program may have benefits that exceed the immediate need for food provision.

Albarran et al. described *Challenges in designing interventions for food insecure families with food allergy in a Californian Latinex cohort.* Therein, the authors reflected on challenges, ranging from undocumented populations, to barriers related to language and digital literacy, and provided thoughtful commentary on how to contribute to solutions. Threaded through all proposed solutions is the need for collaboration and regular communication with these communities, rooted in humility and relationship building.

Unanticipated supply chain disruptions stemming from natural disasters or global pandemics can have a particularly significant impact on families with food allergy. In 2022, market disruptions initially stemming from COVID-19 and compounded by formula recalls and the shutdown of a major manufacturer, culminated in a shortage of infant formula across the U.S. Because specialty formulas were also in scarce supply, infants with cow's milk protein allergy (CMPA) were uniquely affected. Families struggled to find alternative safe and affordable formulas while pediatric healthcare providers tried to make evidence-based recommendations about the safety and efficacy of alternatives. Fabbrini et al. surveyed parents affected by the formula shortage in the original research entitled *Navigating formula shortages: associations of parental perspectives on transitioning to alternative infant formulas for cow's milk protein allergy during the 2022 national formula shortage.* Among parents needing to switch formulas for their child with CMPA during the shortage period, factors such as tolerability, assurance, and safety were overwhelmingly rated as “extremely important” in the decision to select an alternative. Parents switching among amino acid formulas cited the value given the expense as a key consideration in their decision making. First line treatment of CMPA with extensively hydrolysed formulas have led to more cost-effective use of healthcare services when compared with amino acid formulas in the US (3) and UK (4).

Researchers also described data on the same topic from the perspective of pediatric healthcare providers in *Managing cow's milk protein allergy during the 2022 formula shortage: decision-making among pediatric healthcare providers*. Like parents, safety, tolerability, and efficacy were identified as key factors when recommending alternative extensively hydrolysed formulas and one formula rated higher on these factors when compared with two others included in the analysis. Although cost was a consideration evaluated in the paper, pediatric healthcare providers did not rate this factor as highly as other factors. Although safety, tolerability, and efficacy are critical, if families are unable to afford alternative special formulas for their children with CMPA, the health care system has a critical gap in access that needs to be filled.

There is no doubt that food allergy is costly. Our special issue highlights some of these costs, and importantly, also contributes to a theoretical roadmap for working to find solutions. Several of the papers in this issue describe cost considerations under times of turmoil such as the COVID-19 pandemic. Emergency response during natural disasters or economic turbulence leave individuals with food allergy particularly vulnerable. Our special issue raises economic issues for the healthcare system, social safety nets, and emergency preparedness to address the needs of those managing food allergy.
